# Identification and functional characterization of the ZmCOPT copper transporter family in maize

**DOI:** 10.1371/journal.pone.0199081

**Published:** 2018-07-23

**Authors:** Hongling Wang, Hanmei Du, Hongyou Li, Ying Huang, Jianzhou Ding, Chan Liu, Ning Wang, Hai Lan, Suzhi Zhang

**Affiliations:** Key Laboratory of Biology and Genetic Improvement of Maize in Southwest China of Agricultural Department, Ministry of Agriculture, Maize Research Institute, Sichuan Agricultural University, Chengdu, China; Universidade do Minho, PORTUGAL

## Abstract

Copper (Cu) is an essential micronutrient for plant growth and development; Cu homeostasis in plant is maintained by the important functions of Ctr/COPT-type Cu transporters. Although the *COPT* genes have been identified in *Arabidopsis thaliana* and rice, little is known about Cu transporters in maize. In this study, three-members of putative maize Cu transporters (ZmCOPT 1, 2 and 3) are identified. *ZmCOPT* genes have expression in all of the tested tissues, including roots, stems, leaves and flowers (male and female), and their expression levels vary responding to stress due to Cu-deficiency and excess. Functional complementation and overexpression together with Cu uptake measurements in *ZmCOPTs*-transformed *ctr1⊿ctr2⊿*mutant strain or the wild type strain of *Saccharomyces cerevisiae* show that the three ZmCOPT members possess the ability to be Cu transporters. Among these, ZmCOPT1 and ZmCOPT2 have high-affinity while ZmCOPT3 has low-affinity. In addition, ZmCOPT2 tend to specifically transport Cu (I) but no other bivalent metal ions.

## Introduction

Copper (Cu) is an essential micronutrient metal required by plants for growth and development. Cu plays essential roles in many biological processes, such as in photosynthesis, respiratory electron-transport chains, oxidative stress protection, hormone signaling and cell wall metabolism. Cu also acts as a structural element in regulatory proteins and cofactors in many enzymes including cytochrome c oxidaxidase, plastocyanin, superoxide dismutase, and amino oxidase [1]. Cu deficiency in plants induces certain symptoms, such as plant chlorosis (especially in young leaves), defects in cell wall formation and lignification which causes insufficient water transport, and defective pollen development and viability [2]. In biochemical reactions, the utility of Cu depends on the cycle between oxidized Cu (II) and reduced Cu (I) states in enzymes and electron carriers [3]. However, when Cu is in excess, cycling between Cu (I) and Cu (II) can produce highly toxic hydroxyl radicals which subsequently damage macromolecules [4]. Therefore, maintaining the homeostasis of Cu, in which Cu transporters play a pivotal role, is vital for normal plant development.

A number of different types of transporter proteins that can mediate Cu uptake have been reported; the two major transporters are Ctr/COPT-type Cu transporters and P-type heavy metal ATPases [5–8]. Ctr/COPT proteins not only contain multiple protein families in different organisms, they are also highly specific Cu transporters compared with the P-type heavy metal ATPases which can transport a series of ions, such as Cu^+^, Zn^2+^, Mn^2+^, etc. [9]. Results from metal competition experiments have shown that Ctr/COPT proteins predominantly transport Cu (I) (and the isoelectric Ag^+^) but not Cu (II) [10–12]. The dominant form of Cu in soils is Cu (ll) which can be reduced to Cu (I) in the proximal vicinity of the cell and supply Cu (I) to the putative Cu transporter in *Saccharomyces cerevisiae* [13, 14]. Ctr/COPT family members contain three putative transmembrane-spanning domains (TM1-3), an amino-terminal region rich in methionine residues and a carboxy -terminal region rich in cysteine. Among these, TM2 and TM3 are reported to be strictly conserved in Ctr/COPT protein members [15, 16]. There are important signal motifs MxxxM of TM2 and GxxxG of TM3. Especially, methionine residues in the MxxxM motif are essential for Cu transport activity [17]. Based on previous investigations, a structural model has been proposed for Ctr/COPT mediated process of Cu transport. Firstly, the extracellular M-rich motif in the amino-terminal region of the Ctr/COPT protein sequesters Cu (I) to the entrance of the pore, a channel for passage of Cu across the lipid bilayer composed of a set of stacked methionine triads. After passing through the pore, Cu (I) binds to carboxy-terminal Cys-rich motifs, and is then delivered to membrane-associated metallochaperones for targeted distribution [18].

The first Ctr/COPT protein identified in *S*. *cerevisiae* was yCtr1 (ScCtr1), which affected high-affinity Cu uptake [19]; the next two proteins were yCtr2 (ScCtr2) (a low-affinity Cu transporter) and yCtr3 (ScCtr3) (a high-affinity Cu transporter)[20, 21]. Other Ctr/COPT proteins which have been identified including SpCtr4, SpCtr5 and SpCtr6 in fission yeast (*Schizosaccharomyces pombe*) [22, 23]; hCtr1 and hCtr2 in humans (*Homo sapiens*) [24, 25]; mCtr1 in mice (*Mus musculus*) [26]; PtCOPT1-7 in *Populus trichocarpa* [27]; AtCOPT1-6 in *Arabidopsis thaliana* [10, 15, 28]; OsCOPT1-7 in rice (*Oryza sativa*) [29], and VvCTr1 in grapevine(*Vitis vinifera*) [30]. Various yeast Cu transporter mutants, such as ctr1⊿mutant, *ctr1*⊿*ctr2*⊿ mutant and *ctr1*⊿*ctr3*⊿ mutant, have widely been used in heterologous functional complementary system to identify Cu transporters from other species [24, 29, 31]. Among these, COPTs have only been functionally examined in *Arabidopsis* and rice.

In *Arabidopsis*, AtCOPT1, AtCOPT2 and AtCOPT6 could fully restore the growth defects of the yeast *ctr1*⊿*ctr3*⊿ double-mutant strain; AtCOPT3 and AtCOPT5 could only restore growth defects to a modest level. However, AtCOPT4, which defected in the MxxxM motif of TM2, could not complement the growth defect of the same mutant strain [10]. In rice, two kinds of Ctr/COPT-type Cu transporters exist: high-affinity and low-affinity Cu transporters. OsCOPT7 is the only protein which can mediate high-affinity Cu transport by itself [29]. Besides, OsCOPT2, OsCOPT3, or OsCOPT4 can cooperate with OsCOPT6 individually by forming heterologous dimers or polymers tomediate high-affinity Cu uptake. On the other hand, OsCOPT3, OsCOPT4 or OsCOPT6 can mediate low-affinity Cu transport by themselves [29]. In addition, expression of OsCOPT1, OsCOPT5 or XA13, a susceptible protein to pathogenic bacterium *Xanthomonas oryzae pv*. *Oryzae* (Xoo), alone or coexpression of any two of the three proteins could not complement the phenotype of *S*. *cerevisiae* mutant unless coexpression of all of the three proteins [32].

Base on sequence homology, two putative Cu transporters have been mentioned in maize, one of three major global food crops [33]. However, detailed information about the functions of these transporters has not been reported. In our study, we identified and characterized three Ctr/COPT-type Cu transporter genes in maize. The genetic assumption of their ability to transport Cu was performed by functionally complementing the growth defects of the yeast mutant *ctr1*⊿*ctr2*⊿. Cu uptake assays have also demonstrated these Cu transporters have specificity in Cu absorption. Moreover, the expression profile of maize Cu transporters in different tissues and under different Cu concentrations supports further evidence that these transporters are related to Cu transportation.

## Materials and methods

### Plant growth and treatment

Seeds of the maize inbred line 178 were surface-sterilized in 0.5% (w/v) NaOCl for 15 min and then 75% (w/v) ethanol for 2 min before being germinated for 3 d at room temperature on paper soaked with distilled water. The seedlings were then transferred into containers with Hoagland’s nutrient solution [34] and grown in a greenhouse (70% relative humidity, 14 h/28°C and 10 h/22°Cday-night cycle and 300 μmol m^–2^s^–1^ intense luminosity) for about 7 d. The nutrient solution was renewed every two days. To analyze plants for effects of Cu stress, ten-day-old seedlings were grown in a Hoagland’s solution containing 0.5 μM Cu (CuSO4) (Cu excess) or without Cu (Cu deficiency) for 24 h, respectively.

### Identification and characterization of maize COPT proteins

Using the TBLASTP program, the putative COPT gene family was identified from the MaizeSequence database (http://www.MaizeSequence.org), based on six and seven COPT amino acid sequences from *Arabidopsis* and rice, respectively [10, 15, 29]. The preliminary COPT candidates, which were selected with e-values <-10, were further examined using the SMART (http://smart.embl-heidelberg.de/) and Pfam (http://pfam.sanger.ac.uk) databases to ensure the presence of the Ctr domain. Multiple sequence alignment and predictions of transmembrane-spanning domains were performed using DNAMAN (Lynon Biosoft) and TMHMM Server v2.0 (http://www.cbs.dtu.dk/services/TMHMM/), respectively. In addition, by searching the GenBank database (https://www.ncbi.nlm.nih.gov/genbank/), 21 COPT/Ctr protein sequences from rice, *Arabidopsis*, yeast and humans were retrieved for phylogenetic tree establishment by using IQ-TREE(http://iqtree.cibiv.univie.ac.at/)and the neighbor-joining algorithm of MEGA 5.0.Gene IDs of *Ctr/COPT* genes identified from NCBI are listed in S1 Table. Promoter analysis was performed by using database PlantCARE(http://bioinformatics.psb.ugent.be/webtools/plantcare/html/) and the *cis*-elements were listed in S2 Table.

### Gene expression analysis

The expression patterns of maize *COPT* genes in different tissues (roots, stems, leaves, male and female flowers) was detected by semi-quantitative RT-PCR; response to Cu deficiency and Cu excess stress in plant roots and shoots were detected using real-time quantitative RT-PCR. The *ZmCOPT* gene-specific primers were designed according to the cDNA sequences obtained from the MaizeSequence database, these information are listed in S3 Table. Total RNA isolation, subsequent cDNA synthesis, semi-quantitative RT-PCR and real-time quantitative RT-PCR were performed as the methods of Li [35]. *ZmGAPDH* was used as the reference gene in our experiment. Each qRT-PCR or RT-PCR assay was carried out in triplicate in each of the three biological repeats. Statistic analysis was assessed by REST [36].

### Plasmid constructs

Complete ORFs of the maize *ZmCOPT1–3* genes were amplified by using gene-specific primers and cDNA as the template (S4 Table, S1 Text). PCR was then performed using the high-fidelity KOD enzyme (TOYOBO, Shanghai, China). The PCR products were separated on a 1.0% agarose gel and purified using a Gel DNA Purification Kit (Tiangen, Beijing, China). The purified PCR products were then cloned into the pMD19-T vector (TaKaRa, Dalian, China) and verified by sequencing. Subsequently, cDNAs of *ZmCOPT1*, *ZmCOPT2* and *ZmCOPT3* were individually subcloned into *Eco*RI/*Bam*HI sites, *Bam*HI/*Sac*I sites and *Eco*RI/*Sac*I sites of pGADT7 (+Leu) vectors in which gene expression is driven by the constitutive promoter *ADH1*.

### Functional complementation assignment in yeast

*S*. *cerevisiae* wild type BY4742 (genotype: MATα, *his3*⊿, *leu2*⊿, *lys2*⊿, *ura3*⊿) and mutant *ctr1*⊿*ctr2*⊿ (BY4742 background; genotype: MATα, *his3*⊿, *leu2*⊿, *lys2*⊿, *ctr1*⊿:: *KanMX4*, *ctr2*⊿:: *URA3*) of *S*. *cerevisiae* were used to perform functional complementation. The *ctr1*⊿*ctr2*⊿mutant strain lacks Cu transporters due to truncation in the *yCtr1* and *yCtr2* genes and interruption in the *yCtr3* gene [21]. This mutant strain has been noted to grow on a dextrose medium (YPD: 1% yeast extract, 2% peptone, 2% dextrose, 1.5% agar) but can not grow on an ethanol/glycerol medium (YPEG: 1% yeast extract, 2% Bactopeptone, 3% glycerol, 2% ethanol, 1.5% agar) due to the defective mitochondrial respiratory chain caused by the inability of cytochrome C oxidase to obtain its Cu factor [37].

To test Cu transport activities of ZmCOPT members, *ZmCOPTs* yeast expression constructs and the corresponding control vectors were transformed into the *ctr1*⊿*ctr2*⊿mutant and wild cells of *S*. *cerevisiae* using the lithium acetate method [38]. The transformed yeast cells were grown in SC-Leu to OD_600_nm = 1.0 and verified by PCR amplication (S5 Table). The cells were harvested by centrifugation (5,000×g, 2 min) and washed five times with sterile water to remove Cu. Several 10-fold diluted clones of the transformed *ctr1*⊿*ctr2*⊿yeast cells were then plated as drops on YPEG and YPD media; clones of the transformed wild yeast cells were plated as drops on YPD media with a Cu supplement (0 mM, 0.5 mM and 1 mM CuSO4). The plates were incubated for 3 to 6 days at 30°C. The growth rate of the transformed *ctr1*⊿*ctr2*⊿strains was also monitored using a slightly modified method. In brief, these strains were grown in liquid YPEG to log phase (OD_600nm_ = 0.6–0.8). The cells were collected by centrifugation (5,000×g, 10 min) and diluted to an OD_600nm_ of 0.1 to culture in liquid YPEG at 30°C, 160 rpm. The OD_600nm_ was measured every 2 h for the first 12 h and then at 24 h.

### Copper uptake measurement in yeast cells

*S*. *cerevisiae ctr1*⊿*ctr2*⊿ mutant cells harboring plasmids were incubated in liquid YPD to log phase (OD_600nm_ = 0.6–0.8), collected by centrifugation (5,000×g, 10 min), and diluted to an OD_600nm_ = 1.0. CuCl_2_ was then added to 100 ml of the culture to a final concentration of 0.5 μM; cells were incubated at 30°C, 160 rpm for 10 min. To further detect whether the ZmCOPT Cu transporter proteins had absorbed other metal ions, CuCl_2_, AgNO_3_, FeCl_2_ and ZnCl_2_ at a final concentration of 0.5 μM was independently added to the cell culture containing *ctr1*⊿*ctr2*⊿mutant cells harboring the *ZmCOPT2* expression vector and the empty vector (OD_600nm_ = 1.0). The cells were incubated at 30°C, 160 rpm for 10 min. The cell culture was centrifuged (5,000×g, 10 min) to remove yeast; the supernatant was retained and residual metal ion contents were analyzed by flame spectrophotometer (Pride SpectrumFP650, Shanghai, China). This test was independently performed in triplicate at least twice.

## Results

### ZmCOPTs of maize has all the conserved features of known Ctr/COPT-type Cu transporters

To identify the gene family members of *COPT/Ctr* in the maize genome, a systematic TBLASTP search was performed using six known Arabidopsis and seven known rice full-length COPT amino acid sequences. Based on an e-value threshold of 10^−10^ and the typical structural characters of COPT, three non-allelic sequences were identified and named as ZmCOPT1 (GRMZM2G003179), ZmCOPT2 (GRMZM2G042412) and ZmCOPT3 (GRMZM2G317696). The predicted proteins were 157–170 amino acid (aa) residues in length which were separately located on the maize chromosome 7, 3 and 4.

**Fig 1:**
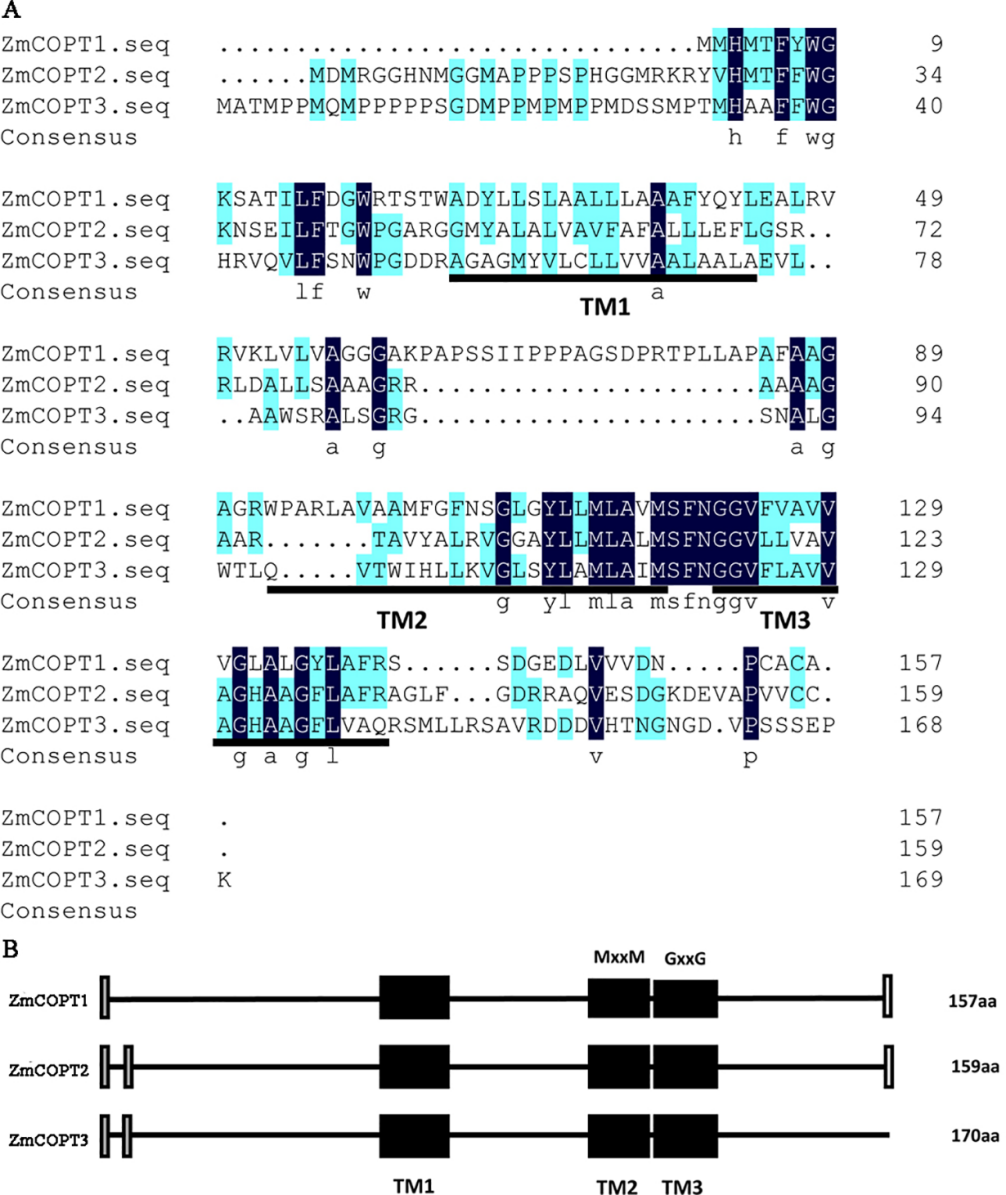
Multiple sequence alignment showing TM regions and the structure of the ZmCOPTs. A: The multiple alignment of protein sequences of ZmCOPT family members was performed using DNAMAN software. Bold lines indicate predicted transmembrane domains and conserved amino acids are shaded in blue. B: The structure of the ZmCOPT proteins: lines represent the protein chains; black boxes represent predicted transmembrane (TM1, TM2, TM3) regions; gray boxes indicate the positions of Met-rich motifs; white boxes indicate the positions of CC or CxC motifs.

The predicted maize ZmCOPTs share an overall hydrophobicity feature of COPT/Ctr transporters: three TMs, an extracellular amino-terminal and a cytoplasmic carboxy-terminal (Fig 1 and S1 Fig). Among these, TM2 is separated from TM3 by only a few residues (Fig 1), and the conserved motif MxxxM in TM2 and GxxxG in TM3(x representing any amino acid) are found. The amino-terminals of ZmCOPTs also have the conserved Met-rich motifs: one in ZmCOPT1 and two in ZmCOPT2 and ZmCOPT3, respectively. Carboxy-terminals, which eventually release Cu into cytoplasm, tend to have Cys-motifs. As expected, the CxC motif was detected in ZmCOPT1, and the CC motif was detected in ZmCOPT2 at the carboxy-terminal. Surprisingly, the Cys-motif has not been found in ZmCOPT3 (Fig 1). In addition, the promoter analysis showed that the *ZmCOPT* genes probably involved in light responsiveness, stress response, phytohormone response, etc (S2 Table). Interestingly, CuRE box, a promoter element related to copper ion transport, has not been found in the promoter region of *ZmCOPTs*. Nevertheless, we have found multiple copy of GTAC motif, the core sequence of CuRE box, in all three promoters of *ZmCOPTs* (S2 Text). SQUAMOSA promoter binding like protein 7 (SPL7) mediates the transcriptional activation of the genes involved in Cu homeostasis, through binding of its SQUAMOSA promoter binding protein (SBP) domain to the GTAC motifs present in the promoters of the target genes. These targets include the high-affinity Cu uptake transporters COPT1 and COPT2 [39].In addition, the promoter region of *ZmCOPT2* contains the AT-rich sequence which is the core sequence of MRE (metal regulatory element) (S2 Table). The potent Cu-responsive *AMT1* gene, which encodes a copper metalloregulatory transcription factor, its autoactivation was elicited by the single MRE harbored in its promoter [40].

**Fig 2:**
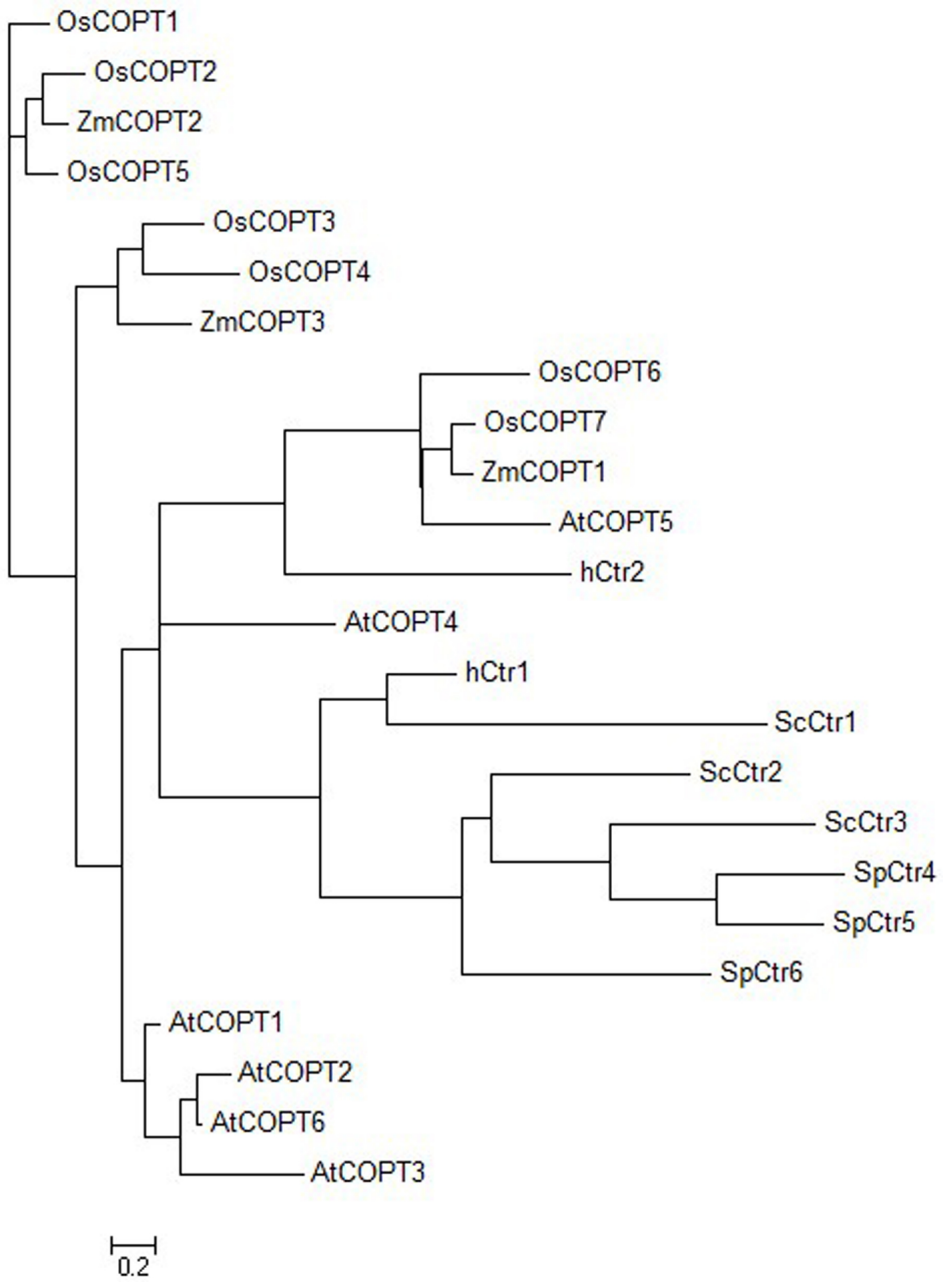
Phylogenetic analysis of maize, rice and *Arabidopsis* COPT members. Phylogenetic tree of the Ctr/COPT family proteins was constructed using the IQ-TREE software. Abbreviations correspond to the following organisms: *Homo sapiens* (*h*); *Saccharomyces cerevisiae* (*Sc*); *Schizosaccharomyces pombe (Sp*); *Oryza sativa* (*Os*); *Arabidopsis thaliana* (*At*) and *Zea mays* (*Zm*).

A phylogenetic tree was further established to examine the evolutionary relationship of 24 COPT/Ctr proteins from various species using the software IQ-TREE (Fig 2). As shown in Fig 2, these COPT/Ctr proteins were classified into four groups. Group 1 included all the members of plant species, seven rice COPTs (OsCOPT1 to OsCOPT7), six *Arabidopsis* COPTs (AtCOPT1 to AtCOPT6) and three maize COPTs (ZmCOPT1 to ZmCOPT3). Group 2 contained the unique member, baker’s yeast (ScCtr1). Group 3 contained two members from humans, hCtr1 and hCtr2. Group 4 included Ctr proteins originated from fission yeast (SpCtr4, SpCtr5, and SpCtr6) and baker’s yeast (ScCtr2 and ScCtr3). Among the group, Group 1 had two main clades and each clade contains representatives from all three plant species. The phylogenetic analysis using the software MEGA 5.0 obtained the similar result (S2 Fig). This result indicates a common ancestor gene existed in each clade before the divergence of monocotyledonous and dicotyledonous plants. ZmCOPTs are evolutionarily closely related to plants originated from *Arabidopsis* AtCOPTs and rice OsCOPTs rather than those of other species. Meanwhile, ZmCOPTs are more closely related to OsCOPTs than to AtCOPTs. For example, ZmCOPT1 is clustered together with rice OsCOPT7, ZmCOPT2 with OsCOPT2, and ZmCOPT3 with OsCOPT3 and OsCOPT4 (Fig 2); this is consistent with the close evolutionary relationship between maize and rice.

### The expression patterns of *ZmCOPT* genes were influenced by Cu stress conditions

**Fig 3:**
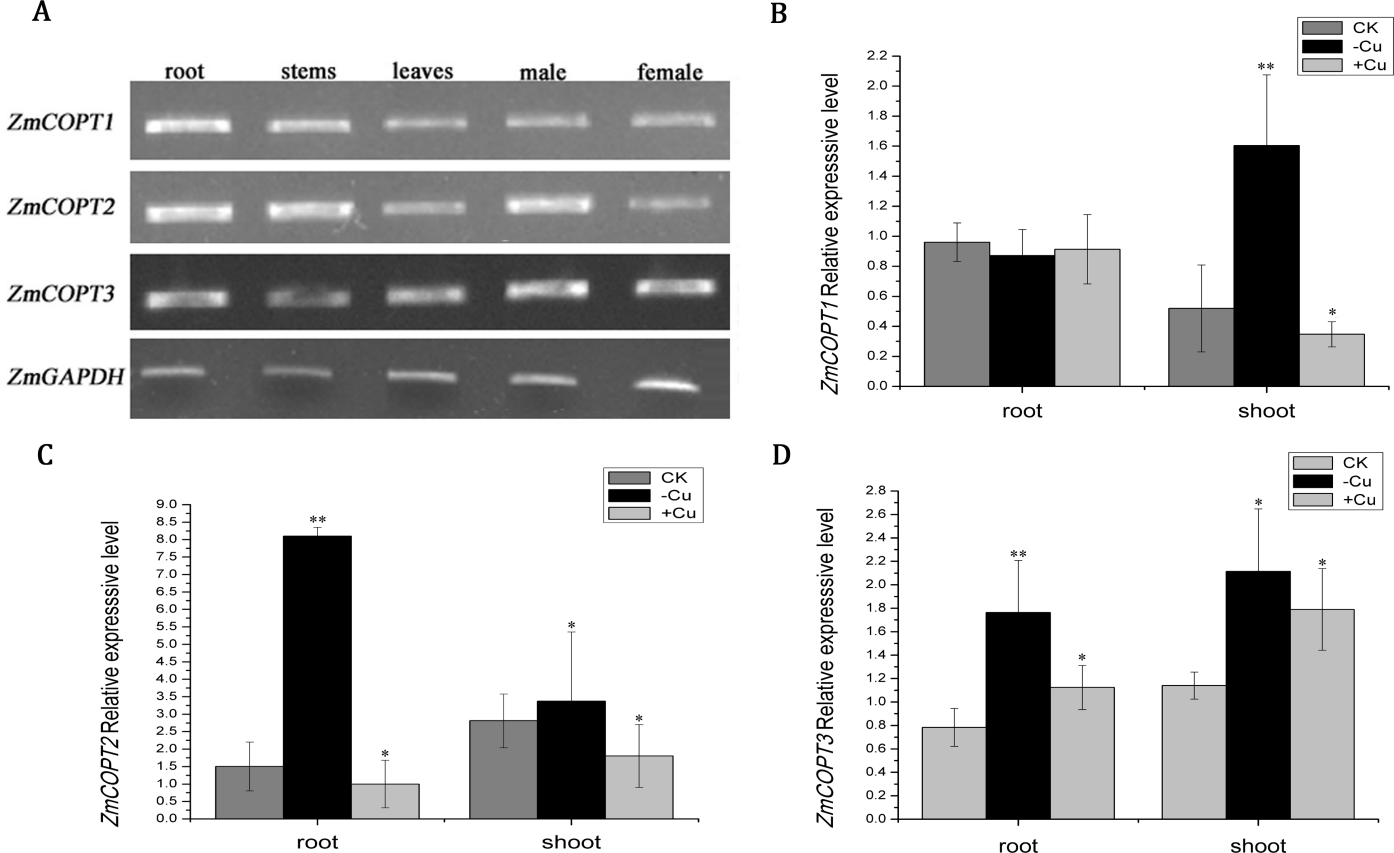
Expression patterns of the three *ZmCOPT* genes in different tissues with different copper status in maize. A: Tissue expression pattern of *ZmCOPTs* measured by semi-quantitative RT-PCR, with *ZmGAPDH* as reference genes. Total RNAs were prepared from the indicated tissues. B, C and D: Transcript levels of the genes in the roots and shoots were quantified at 0 h (CK) and 24 h after Cu deficiency or 0.5 μM Cu treatment by Real-Time quantitative PCR. Relative gene expressions were normalized to the *ZmGAPDH* gene. The error bars represent ± SD. The sign"*”and “**”presented as statistically significant difference at P < 0.05 and at P < 0.01 level (REST).

To learn more about the function of ZmCOPTs, the expression pattern of the three *ZmCOPT* genes was examined. As shown in Fig 3A, *ZmCOPT* genes were expressed in all of the tested tissues, including roots, stems, leaves and flowers (male and female). To further characterize the roles of potential Cu transport ZmCOPT in maize plants, the expression of *ZmCOPT* genes in both roots and shoots under stress conditions (Cu-deficient or Cu-excess) were investigated. The expression of the *ZmCOPT1* gene was strongly up-regulated under Cu-deficient conditions whereas it was suppressed by Cu-excess stress in shoots; results from roots indicated no significant variation due to Cu-deficient or excess. The expression level of *ZmCOPT2* was stimulated by a deficiency of Cu and suppressed by an excess of Cu in both shoots and roots. Interestingly, the expression of *ZmCOPT3* was induced in both shoots and roots regardless of the Cu levels (deficient or excess). These results reveal that the expression levels of *ZmCOPT* genes respond to some extent by an excess/deficiency of Cu in the environment.

### Cloning of *ZmCOPT* genes and functional complementation in the *ctr1*⊿*ctr2*⊿ mutant of *S*. *cerevisiae*

**Fig 4:**
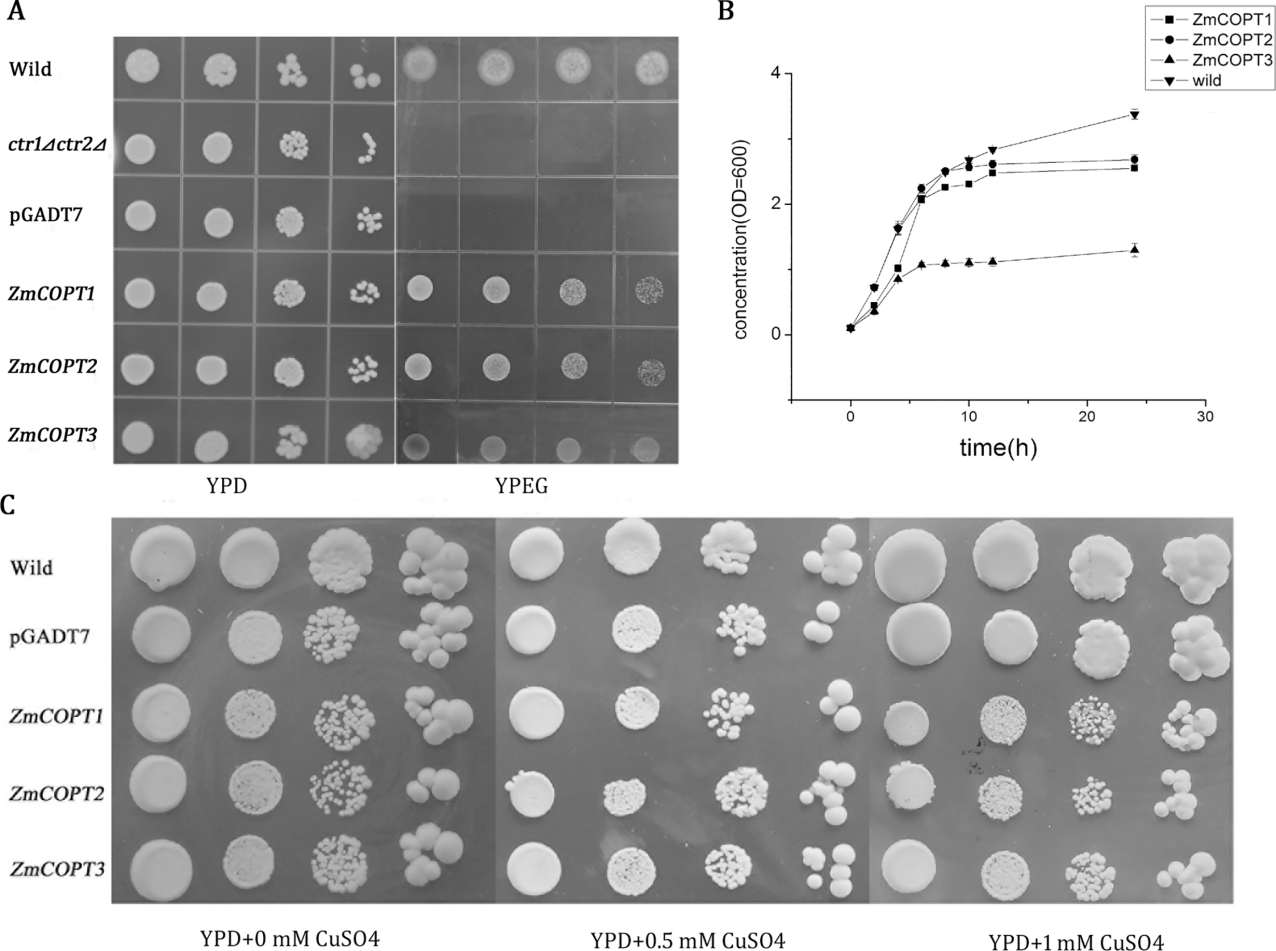
Complementation of the *ctr1*⊿*ctr2*⊿ *S*. *cerevisiae* mutant by *ZmCOPT* genes on YPEG media and the growth patterns of the wild yeast cells transformed with *ZmCOPT* genes on YPD media with excess Cu. A: *ctr1*⊿*ctr2*⊿ mutant was transformed with vector pGADT7 (negative control) or with *ZmCOPTs* expression vector. Wild yeast cells were positive control. Cells were grown on YPD or YPEG media for 3 to 6 days at 30°C. B: The density of cells containing *ctr1*⊿*ctr2*⊿mutant transformed with *ZmCOPTs* and wild yeast cells in liquid YPEG (OD_600_) was monitored every 2 h for the first 12 h and at 24 h. Data are average values of three independent experiments and bars indicate mean ± SD. C: The growth patterns of the wild yeast cells and wild yeast transformed with *ZmCOPTs* expression vector or empty vector pGADT7 on YPD media supplemented with Cu (0 mM, 0.5 mM, 1 mM CuSO4) for 3 to 6 days at 30°C.

To investigate the potential role of the three ZmCOPTs in Cu transport, the complete cDNA of these *ZmCOPT* genes were amplified by gene-specific primer and confirmed by sequencing (S3 Table, S1 Text and S3 Fig), and then individually subcloned into the yeast expression vector pGADT7 and verified by PCR amplication (S4 Fig). The complementation assay was performed using the *ctr1*⊿*ctr2*⊿*S*. *cerevisiae*mutant; this mutant lacks the Cu transporters Ctr1 and Ctr2 and accordingly cannot grow on YPEG media [37]. The *ZmCOPTs* expression vectors and the empty vector pGADT7 were transformed into *ctr1*⊿*ctr2*⊿*S*. *cerevisiae* mutant. Among these, the wild type strain BY4742 of *S*. *cerevisiae* was used as the positive control while the *ctr1*⊿*ctr2*⊿ mutant strain and the *ctr1*⊿*ctr2*⊿ mutant strain transformed with the empty pGADT7 vector were used as the negative controls. As shown in Fig 4A, when the three *ZmCOPT* genes were individually transformed, the growth defect of the *ctr1*⊿*ctr2*⊿ mutant was restored on the YPEG media. Results showed that ZmCOPT1 or ZmCOPT2 could efficiently rescue the growth defect of *ctr1*⊿*ctr2*⊿ mutant while the complementary effect of ZmCOPT3 was weaker (Fig 4A). To further assess the complementation of each *ZmCOPT* gene in *ctr1*⊿*ctr2*⊿ mutant strains, the growth rate of wild type strains and all transformed mutant strains in liquid YPEG were also monitored. As shown in Fig 4B, the ctr1⊿ctr2⊿ mutant strains transformed with *ZmCOPT3* grew more slowly than wild type strains and those strains transformed with *ZmCOPT1* or *ZmCOPT2*; *ZmCOPT3* therefore reached the stationary phase before *ZmCOPT1* or *ZmCOPT2*. This result is consistent with the weak rescuing phenotype of ZmCOPT3 observed on agar plates, suggesting that both ZmCOPT1 and ZmCOPT2 can mediate high-affinity Cu transport in maize whilst ZmCOPT3 can mediate low-affinity Cu transport.

### Overexpression of *ZmCOPT* genes conferred growth retardation of wild type *S*. *cerevisiae*

Excessive copper uptake will be toxic to the cell. On YPD media with and without Cu, an attempt to clarify this suggestion was undertaken by monitoring the growth of wild type *S*. *cerevisiae* and those (wild type background) individually transformed with *ZmCOPT* genes. As shown in Fig 4C, the growth of wild type strains and those transformed with *ZmCOPTs* had a similar phenotype without Cu or with the addition of 0.5 mM Cu on YPD media. However the growth of ZmCOPTs overexpressed yeast strains with the addition of 1 mM Cu were severely inhibited compared with the wild type yeast strains. These results firmly support genetic evidence that ZmCOPTs necessarily participate in the process of Cu transport.

### Copper uptake measurements in the *ctr1⊿ctr2⊿* mutant of *S*. *cerevisiae*

**Fig 5:**
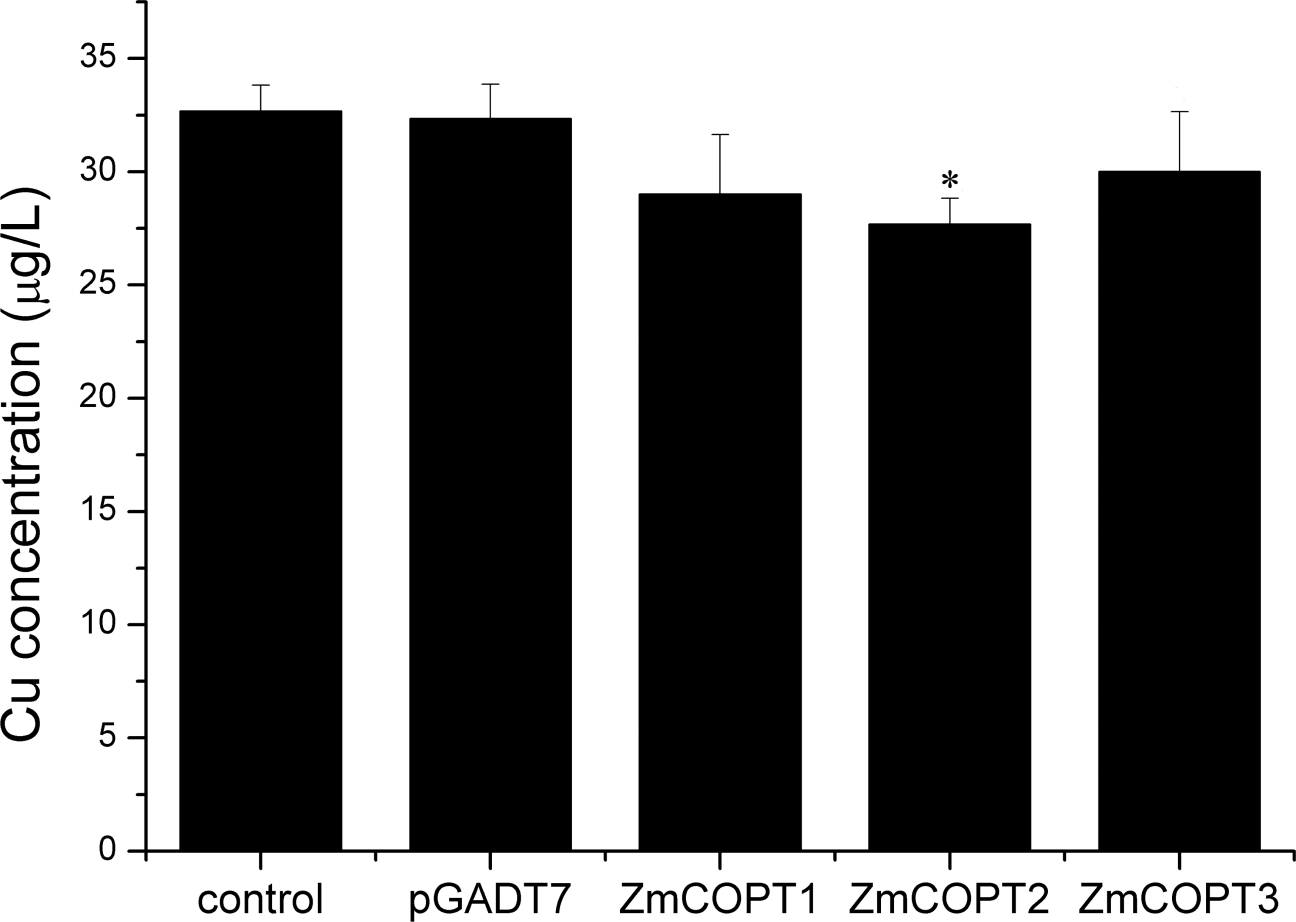
Copper uptake measurements in *S*. *cerevisiae* cells. ZmCOPT1, ZmCOPT2 and ZmCOPT3 restore the copper transport defects of the *ctr1⊿ctr2⊿* yeast mutant strain. Copper content was measured in supernatant of the mutant transformed with *ZmCOPTs* expression vector or the empty vector pGADT7, and copper content in liquid YPD without yeast cells as a control. The error bars represent ± SD. The sign"*”and “**”presented as statistically significant difference at P < 0.05 and at P < 0.01 level (student *t*-test).

**Fig 6:**
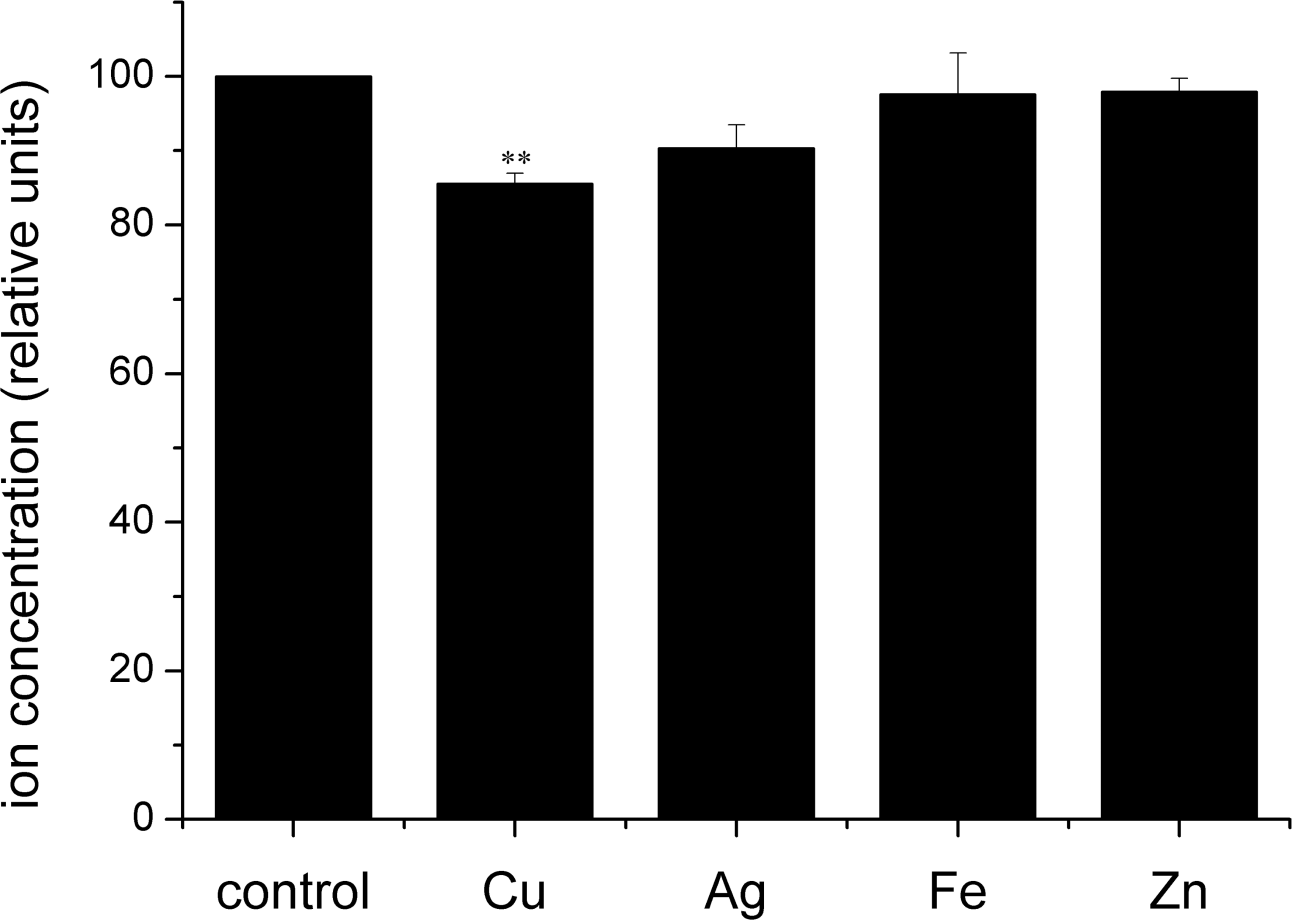
The ZmCOPT2 showed transport specificity to Cu. Copper, silver, iron and zinc content were measured in supernatant of the *ctr1*⊿*ctr2*⊿ yeast mutant transformed with *ZmCOPT2* or the empty vector pGADT7. Ion content was compared to control cells *(ctr1*⊿*ctr2*⊿yeast mutant transformed with pGADT7) represented as 100% (control bar). The error bars represent ± SD. The sign"*”and “**”presented as statistically significant difference at P < 0.05 and at P < 0.01 level (student *t*-test).

To further evaluate the Cu transport function of the maize ZmCOPTs, Cu uptake assays were individually carried out in *ctr1*⊿*ctr2*⊿mutant strains transformed with *ZmCOPT1*, *2* and *3*. Cu content obviously decreased in the supernatant of *ZmCOPT*-transformed yeast strains compared with those transformed with the empty vector (Fig 5). Consistent with our growth complementation results, Cu uptake was much higher in *ctr1*⊿*ctr2*⊿mutant strains transformed by *ZmCOPT1* or *ZmCOPT2* than by *ZmCOPT3* (Fig 5). Despite Cu, the content of other metals, such as iron, silver and zinc, were also measured to verify the transport specificity of ZmCOPTs. ZmCOPT2 was selected for the analysis as it was the highest-affinity Cu transporter of the three maize genes. The results demonstrated that in the supernatant of *ZmCOPT2* transformed *ctr1*⊿*ctr2*⊿ mutant strains, there was only a slight decrease in iron or zinc content against a distinct decline in Ag content (9.65%) and Cu (14.41%) in comparison with the control (Fig 6). Taken together, these results indicated that restoration Cu uptake defects via expression of *ZmCOPT1*, *2* and *3* are coincidently with growth defects. The results also show that ZmCOPT2 tend to specifically transport Cu (I) (and Ag) but no other bivalent metal ions.

## Discussion

Cu is involved in a range of important biological processes which makes it essential for normal plant growth and development. Although *COPT* genes encoding Cu transporter proteins have been identified in several plant species, their function has only been reported in *Arabidopsis* and rice [10, 15, 29]. In this study, we report the identification and characterization of three members of the *ZmCOPT* gene family in maize.

ZmCOPTs share a conserved structure with Cu transporter homologs from rice and *Arabidopsis*. For example, each ZmCOPT contains three TMs and the conserved residues were largely restricted to the three TMs, giving the clue of functional conservation of these ZmCOPTs. In addition, as each TM2 of the *ZmCOPT* genes contains the MxxxM motif, this suggests ZmCOPTs are potential Cu transporters. The MxxxM motif is essential for Cu uptake and may coordinate Cu during intramembraneous transport [17]. Substitution of the methionine of TM2 with alanine or serine in yCtr and hCtr proteins resulted in the proteins losing the ability to transport Cu [17]. And AtCOPT4 protein which does not possess the MxxxM motif is also incapable of restoring the growth defect of *ctr1*⊿*ctr3*⊿ mutant of *S*. *cerevisiae* [10]. Furthermore, all three ZmCOPTs have the Met-rich motif in their amino-terminal which can sequester Cu (I) to the Cu transport channel, but the number of this motif varied. ZmCOPT1 and ZmCOPT2 also contain the Cys-rich motif, a motif which controls the release of Cu into the cytoplasm; this motif is not present in ZmCOPT3. In addition, GTAC motif and the AT-rich element found in the promoter region of ZmCOPT2 were related to Cu transport [39,40]. These conserved structural features of ZmCOPTs strongly suggest a functional involvement in Cu transport; variation however may occur among the ZmCOPTs to enable them to adapt to fluctuating environmental variation of Cu. The fact that ZmCOPTs clustered together with COPTs originating from plants in the same group of the phylogenetic tree provides further proof that ZmCOPTs are Cu transporters.

Generally, the expression pattern of a gene is closely correlated with its function. In our investigation, the three *ZmCOPT* genes had displayed a constitutively expression pattern in all of the detected tissues, but the expression level are not the same for all organs (Fig 3), suggesting that maybe each ZmCOPT has its function in a specific part of the plant. This expression pattern has also been reported for the COPT transporters in *Arabidopsis* (except *AtCOPT6*), rice and grapevine [10, 29, 31]. On the other hand, Zm*COPT* genes had a distinct response under Cu stress conditions. For example, the expression of *ZmCOPT1* in shoots and *ZmCOPT2* in shoots and roots was induced by a deficiency of Cu and suppressed with an excess of Cu; *ZmCOPT3* was induced in both shoots and roots regardless of Cu-deficient or Cu-excess (Fig 3). In the same case, the expression of *AtCOPT2* was induced under Cu-deficient conditions and the expression of *AtCOPT1* and *AtCOPT2* were suppressed with an excess of Cu [10]. And the expression of *OsCOPT1*, *2*, *5* and *7* was induced under Cu-deficient conditions and suppressed with an excess of Cu in rice [29]. An exploring with promoter-fused GUS in plant under Cu deficiency (using Cu chelator) could give more information of the expression pattern of *ZmCOPT* genes.

Previous investigations have reported that a series of Ctr/COPT-type Cu transporters can complement the phenotype of *S*. *cerevisia*e *ctr1*⊿*ctr3*⊿ mutant, these include mCtr1 of mice [26], hCtr1 of humans [24], AtCOPT1, 2, 3, and 5 of *Arabidopsis* [10, 41], and OsCOPT7 of rice [29]. In our investigation, all of the three maize *ZmCOPT* genes also demonstrated their function in Cu transport by genetic complementation of the *ctr1*⊿*ctr2*⊿ mutant. Measurement of Cu content in the corresponding transformed mutant strains was in accordance with the function of ZmCOPTs in Cu transport and the complementation test (Figs 4A and 5). On the contrary, the growth of *ZmCOPTs*-transformed wild type strain was inhibited by excess Cu, the result is similar to the severe growth retardation of yeast strains transformed with the Cu transporter gene *TaCT1* in the presence of 0.5 mM Cu [42], thus providing additional supporting evidence for the ability of ZmCOPT for Cu transport.

Interestingly, the growth rate of *ZmCOPT3*-transformed mutant strains was considerably lower than that of *ZmCOPT1*- or *ZmCOPT2*-transformed mutant strains, whether in solid or liquid media, suggesting a different affinity of these ZmCOPTs in the process of Cu transport: ZmCOPT1 and ZmCOPT2 had high-affinity and ZmCOPT3 had low-affinity. This finding seems to be partially attributed to the variation in number of Met-rich motifs in the amino-terminal of the ZmCOPTs. High-affinity Cu transporters with two Met-rich motifs had a higher ability for Cu transport than those with one [10, 15]. And deletion of the eight Met-rich motifs of yCtr1 decreased copper uptake rate by one-third compared with the wild type [17]. In our study, the high-affinity Cu transporter ZmCOPT2 which had two Met-rich motifs in the amino-terminal seemed transport more Cu than ZmCOPT1, which only had one Met-rich motif, according to the growth inhibition. However, Met-rich motifs could not be used as a measuring criterion of the affinity of Cu transporters. Otherwise, *ZmCOPT3*-transformed wild type and mutant strains of *S*. *cerevisiae* would have a higher Cu transport ability or Cu content than *ZmCOPT1*-transformed. Actually, ZmCOPT3 (with two Met-rich motifs) had the weakest ability to transport Cu among the three ZmCOPTs. Similarly, the high-affinity Cu transporter yCtr3 contains no Met-rich motifs and OsCOPT7 has one motif in the amino-terminal; low-affinity Cu transporters OsCOPT3 and OsCOPT4 have three motifs [8, 29]. Results for phylogenetic analysis showed that ZmCOPT1 was more related to rice OsCOPT7, this being the only gene which could mediate high-affinity copper transporter alone; ZmCOPT2 was more related to rice OsCOPT2, which could cooperate with OsCOPT6 to mediate high-affinity Cu uptake and ZmCOPT3 is more related to rice OsCOPT3 and OsCOPT4, which could mediate low-affinity Cu transport in rice [29]. As to the low-affinity of ZmCOPT3, at least two factors should be taken into account. Firstly, because the Cys-rich motif in the carboxy-terminal of yeast Ctr1 is dispensable for high affinity uptake, the lack of the Cys-rich motif in carboxy-terminal region of ZmCOPT3 suggests that this does not affect its low-affinity to Cu. Secondly, *ZmCOPT3* has the special up-regulated expression pattern whether subjected to Cu-deficiency or Cu-excess stress, perhaps caused by a lack of regulation domain (the Cys-rich motif) in the carboxy-terminal which prevents excessive uptake of Cu [43]. These factors, together with the low-affinity of Cu transport of ZmCOPT3, suggested that it may play a role in basal level Cu transport in a fluctuating environment. Otherwise, *ZmCOPT3* might be controlled by the feedback regulation of biological requirement in maize.

Furthermore, the ZmCOPT2 protein is a high-affinity transporter with specificity for Cu (I), but not for other bivalent ions, such as Mn or Zn, similar to *Arabidopsis* COPT1 and other Ctr1 family members (Fig 6) [10, 11].Moreover, like hCtr1, ZmCOPT2 could also transport Ag due to similar isoelectronic structure of Ag (I) and Cu (I) which can cause competition [11, 12]. So far, COPT/Ctr proteins function specifically in Cu transport. Though the endogenous iron, manganese, or zinc may influence Cu homeostasis by influencing the expression of COPTs in rice, none of them could restore the Fe- or Zn-uptake functions of the yeast mutants [29]. Thus, just like other COPTs, whether or not, the ZmCOPT1 and ZmCOPT3 also show specificity in Cu transport, is required further study to determine. Meanwhile, the direct assessment of Cu content in the ZmCOPTs-transformed yeast cells and conduction the subcellular location study in plant cells and promoter analysis in plant system would also be powerful to explain the Cu transport ability of the ZmCOPTs in the future.

In conclusion, our investigation identified three ZmCOPT transporters and suggested that *ZmCOPT* genes may play essential roles in the uptake and translocation of Cu and that they respond to fluctuating environmental levels of Cu. However, the precise roles of individual *ZmCOPT* genes require further research.

## Supporting information

S1 Text The nucleotide sequences of the three *ZmCOPT* genes. (DOCX)

S2 Text The nucleotides of the promoter sequence of the three *ZmCOPT* genes. (DOCX)

S1 Table *Ctr/COPT* genes identified in some species. (DOCX)

S2 Table The *cis*-element of the promoter sequence of *ZmCOPTs* is predicted by database PlantCARE. (DOCX)

S3 Table PCR primers used for the semi-quantitative RT-PCR and the real-time quantitative PCR. (DOCX)

S4 Table PCR primers used for complete CDS amplification. (DOCX)

S5 Table PCR primers used for *ctr1⊿ctr2⊿S*. *cerevisiae* mutant complementation assays. (DOCX)

S1 Fig The predicted TM regions of the three ZmCOPT proteins. The TM regions of the ZmCOPT proteins were predicted using TMHMM2 (http://www.cbs.dtu.dk/services/TMHMM) 2.0: the red peaks indicate the predicted transmembrane domains of the proteins.
(TIF)

S2 Fig Phylogenetic tree of the Ctr/COPT family proteins constructed by using Mega 5.0. (TIF)

S3 Fig PCR amplification of the entire ORFs of the three *ZmCOPT* genes. Root or leaf cDNA was used as the template. The PCR program contained 35 cycles and genespecific-primer pairs were used.
(TIF)

S4 Fig PCR amplification of *ZmCOPT* genes in yeast cells on the selective medium. (JPG)
