# Supplementary material for: Identification and functional characterization of the ZmCOPT copper transporter family in maize
Source: PLoS One. 2018 Jul 23;13(7):e0199081. doi: 10.1371/journal.pone.0199081 (PMC6056030; doi:10.1371/journal.pone.0199081)
Supplement: S1 Text — (DOCX) [file pone.0199081.s001.docx]

Supplementary Text 1 The nucleotide sequences of the three ZmCOPT genes from sequencing.

ZmCOPT1:

ATGATGCACATGACCTTCTACTGGGGCAAGTCGGCCACGATCCTGTTCGACGGCTGGCGCACCTCCACGTGGGCCGACTACCTTCTCTCCCTCGCGGCCCTGCTCCTCGCCGCCGCTTTCTACCAGTACCTCGAGGCCCTGCGGGTGCGCGTGAAGCTCGTCCTCGTCGCGGGAGGAGGCGCAAAGCCAGCCCCCTCCTCCATCATCCCGCCACCCGCCGGCTCCGACCCGCGGACGCCGCTTCTCGCGCCCGCCTTCGCGGCCGGCGCCGGGCGCTGGCCGGCGCGCCTGGCCGTGGCCGCGATGTTCGGGTTCAACTCCGGCCTCGGCTACCTGCTCATGCTCGCCGTCATGTCGTTCAACGGCGGCGTGTTTGTCGCCGTCGTCGTGGGCCTCGCGCTCGGGTACCTCGCGTTCCGCAGCAGCGACGGGGAGGATCTCGTCGTCGTCGACAACCCCTGCGCCTGCGCCTAA

MMHMTFYWGKSATILFDGWRTSTWADYLLSLAALLLAAAFYQYLEALRVRVKLVLVAGGGAKPAPSSIIPPPAGSDPRTPLLAPAFAAGAGRWPARLAVAAMFGFNSGLGYLLMLAVMSFNGGVFVAVVVGLALGYLAFRSSDGEDLVVVDNPCACA (157aa)

ZmCOPT2:

ATGGATATGAGAGGAGGGCACAACATGGGCGGCATGGCACCGCCGCCGTCCCCTCACGGCGGCATGCGGAAGCGCTACGTCCACATGACCTTCTTCTGGGGCAAGAACTCGGAGATCCTCTTCACGGGGTGGCCCGGCGCGCGCGGCGGCATGTACGCGCTGGCGCTCGTCGCCGTCTTCGCGTTCGCGCTCCTGCTCGAGTTCCTGGGCTCCCGCCGCCTGGACGCGCTCCTCTCCGCCGCGGCCGGCCGGCGCGCGGCAGCGGCGGGGGCGGCGCGCACGGCGGTGTATGCCCTGCGCGTGGGTGGGGCGTACCTGCTCATGCTGGCGCTCATGTCGTTCAACGGCGGCGTGCTCCTCGTTGCAGTCGCGGGCCACGCCGCGGGCTTCCTGGCGTTCAGGGCCGGCCTGTTCGGCGACCGGCGGGCGCAGGTGGAGAGCGACGGCAAGGATGAGGTCGCGCCGGTCGTGTGCTGTTAG

MDMRGGHNMGGMAPPPSPHGGMRKRYVHMTFFWGKNSEILFTGWPGARGGMYALALVAVFAFALLLEFLGSRRLDALLSAAAGRRAAAAGAARTAVYALRVGGAYLLMLALMSFNGGVLLVAVAGHAAGFLAFRAGLFGDRRAQVESDGKDEVAPVVCC (159aa)

ZmCOPT3:

ATGGCGACGATGCCGCCGATGCAGATGCCGCCTCCACCGCCCTCCGGTGACATGCCGCCCATGCCGATGCCGCCTATGGACAGCAGCATGCCCACGATGCATGCGGCCTTCTTCTGGGGCCACCGGGTGCAGGTGCTCTTCTCCAACTGGCCGGGCGACGACCGCGCCGGCGCCGGGATGTACGTCCTCTGCCTCCTCGTGGTGGCCGCGCTCGCCGCGCTCGCCGAGGTGCTCGCGGCATGGTCCCGCGCCCTCTCCGGCCGTGGCTCCAACGCGCTGGGGTGGACGCTGCAGGTCACGTGGATCCACCTGCTGAAGGTGGGCCTCTCCTACCTGGCGATGCTGGCCATCATGTCCTTCAACGGCGGGGTTTTCTTGGCCGTCGTGGCTGGCCACGCCGCCGGGTTCCTCGTTGCGCAGAGATCGATGCTACTACGCTCTGCGGTCCGTGACGACGACGTGCACACCAACGGTAACGGTGATGTCCCTTCGTCGTCAGAACCAAAGCCTTGA

MATMPPMQMPPPPPSGDMPPMPMPPMDSSMPTMHAAFFWGHRVQVLFSNWPGDDRAGAGMYVLCLLVVAALAALAEVLAAWSRALSGRGSNALGWTLQVTWIHLLKVGLSYLAMLAIMSFNGGVFLAVVAGHAAGFLVAQRSMLLRSAVRDDDVHTNGNGDVPSSSEPKP (170aa)
