# Supplementary material for: Identification and functional characterization of the ZmCOPT copper transporter family in maize
Source: PLoS One. 2018 Jul 23;13(7):e0199081. doi: 10.1371/journal.pone.0199081 (PMC6056030; doi:10.1371/journal.pone.0199081)
Supplement: S2 Text — (DOCX) [file pone.0199081.s002.docx]

COPT1

TGTGTGCTCTTGCCAAGTCGCCCTCGTGCAATGCTTCCAGGAATCACTCACTGCTGCTCG

TCCAGCACCAGTTGCTTTTTCTCCACCAAATACTCCACCGTTTTCAGCACCACAAGGATT

TATTCGGTTATTTCTATCCAAATAGATTAGATAAGAATAGAAAAAATTATCACGCTCTGT

TCGGATTATTGGAATTGAATTTCATTCTAATAATAATAATTTAGGCATATATCAATGAAG

TTAATTTGATTTTATGCAAAATATATTTGTATACTATTATAGAAAGATGTCGGAGATATT

TATGTGCTACATTTTTACTGTAGAGGAGTGAGACAAAGAGTGTCATGTAAGTTACAGAGT

AGAAACAAATTCTACTAATTATAAAACCATTTCCCATCCTCTAACCCATGAATTTATGAT

AAACTTATATCTAAACTTTAGAAAGTGGTGGAATGTCAAATTCAAAACTAAATAAGTTAT

TTTTTTTGAGTGAATTCCAATTCCTCTAAAATAAAGGGGTTCAAACGCCCCGTTAGAGAT

TTTGATTTGCTATGGATTTAAATTCACTCAATCCCCTATAATCTATATGAATTGAGATGA

AAACAAATATGCCCTTCGAGCAACTGCAAAAGGGCATCAAAAAATTGTTCCCCAGAACAT

CTTACGGGACTGCTGTTAAAAATCATTCTCTGAAAAGGATAGACTGCTAATAAACTAACA

TCAAACATTTTTGAAACACTACCGTGGCCTGATATTGGGCCGGTAGGCGTCCACCGTGAG

CCGAGACAATTAGAAAAGATGGATACTAAATATCTAAATTTAATATTTTAATATAGGTAT

GCATAGAATTTGATTAATCATATTATTAAAAATATTATGAAAGATAAGAATAAAACATTA

TATAACTTTTTTGTTCATTATTTTTCATACAAAAAATAAAATAAAAAAGCATTCGAATCT

AAATTTGTATGCGTAACCAAATTTTATATCAAGTATTTAAAAAGATATAAAACAAATTTG

ATTATCTATTTATTATGAATCTTAATAAGTTTAATTCCAAAAATAAGAATATAAATTTTC

ATAGCAATTTTATATATTATTTGTTGGCAATTTGAAAGAAGAAAAAAATTAATATCTAAA

TAAATATCCGGATTCATCCTTGTTTTTGCACGGTGGGAATGGGACGTGGGGTGCGCCATT

TGCCGCAAGCGATTTGTGGTCGGATTGGACGTTCGTCTGAACAAACCTTCCACGCGCGGT

TTCTTGGATCGGATTCGATGCGCAGCCTGCCGGCTCCGCTCGGTCAGCTCACATCTAACA

GCTCCACTGGCGCACGTCCTCTGTCCTACTCTGCTCGCCACTGTTTCTTAAGATACACCA

AATAAAAAAAATATAAGAGGAGCCGTTTTACCAAACGATTTATAAAAATACACTGACTTC

TTAGGAAGAGCTGCTCTTATCGAGAAGCTAGAGCCGGAGTCCTTTTTAGAGGAGCCAGAG

CCCTACCAAACAGGGCCTAAGAAAAACCAGCTATTTTGACTGTTATATTTAGATTTTCCT

CCGTTTTGTTTTAGTTATCGCTGAATAGTGCAAAATTTAACTATCCAGCAACAATTAAAA

AAAACGGAGGAAGTAATATATAAGGACACACGGTTGAAATGATAAAAGTCGTTACAGATT

AAAGACAAACGAACAGCGAGAATGACCTCAGTTTCGACCCTATCTTCCTCGACGGGCACG

ACGAAACAACGCAACCGAGGTGGAGCCTCTAACGAATTCGTCTGTGCACGCATCGCTCCA

AAGCGGGGCTGAGTTCCGACTTGTCCAGCTTCCTTTCAGGCTTTCAGCCGGCCGAGCGGT

TGGCGCCCGGCAGGCGGTAGTACAGGACTGTCTACAACTGCAGTACAACAGGTCGGGTCG

TGCGACTGTACTGCGGGCGGCTCGCAAAGCACAGGAGCAGGCCTCGATTAAAAATCTCAC

CGGTCTCTTTCTCTTTCCTCGCCGGCAATCCTTCTCCTCCCTCCCATTCCCATTCCCATC

GCCGCTTCCAAGTCCCAACCGTTCCCTTCTCCGCCCTCGCCGCCGGCCAAC ATGATGCAC

COPT2

ACATAAATACCTACTCCAGCTGTCCTAAATTAAAATTTGTTTTAAACTCTTAATAGATTC

ATACAATAATTAAGTAAAGTTTGTTTTAAACTCTTAATAGATTCATACAATAATTAATGT

ATGTATTTTGTATATATGTCTAGATTAATCACCATCTATTTGAATATAGTCATAAAAATG

AAGTCTAAAACGAATACTATTTTAGGACGGATGGAGTACATTATTTGTGTAACCCACTTT

GCTACCAATATACTCTCTTGTCTTGAAATAGATGATACTTTAGCTATAAATATGTACAGA

TATATCGCTGTTCAGTTTCATTGATAAAATATCATCTATTTTTAGAACGGAGGAAGTGCT

CTCAATGCTTATATTTATATAATATATCATGTTTAATTTGCCCTAATGCACGTATCATCA

GACCATACAATTGTGCACATTTGTATTAGGTTTGTCCTAAGTCGCACTAGTAGAAAAAGG

TTCAACGCCTGTGGGACGATATATTTTTACAGACGTATCCGGTTATCCACCGACTATGCT

ATTTTTAGTGGCGGTTTCTTAAGAAAACCGCCACTAGAAATCGTATTTTTAGAGGCGGTT

CCTTAAGAAAACCGCCAATAGAAATCCATGATTTCTAGTGACGGTTTTCTTAAGGAACCG

CCTATAGAAATCGATTTCTAGTAGCGGTTTTCTTAAGGAACCGCCACTAAAAATCATTTT

TATCCTTAATTTTTCGAGTTTTTCAAACGACCTCGTATGACAAAACCACCAAAATAAAAG

TTGTAGATCTCTAAAAGTTATGAAACTTTGTAGTTGACAACTTTTTTATTTGAACTCATT

TCGGTTCTCAAAAATTGAATCTAAGTATGTCAAATTTAAAATTCAAATTTTGCAAACTAA

CTCGGATGAAAAAAGTGTCGAAATAAAAGTTGTAGAACTTCAAAAGTTATTTAACTTTGT

AGTTGACAACTTTTTTATTTGAATTCGTTTAGGGTCTCAAATAAGCAATTTACACTCAAA

TGGTTGTAATATGTGGACAAAACAACTACAAACTAGACACAAAGTATGTCATAGACGGAG

TGGTAATGGAGGGTACACGCGAGGGTGAGGTCAAAGGTTCGATTACTAACAACCACGTAG

CTTTGCGCGAAAAATGCCGTGACTTGCGACTTCGACTGAGGCCCCCTATTTTTTACTATT

TTTAAACTCTGTTTTATGTTTCCTGGAAACGATTTGCACTGCCGGTTTTATTACGTCGAC

CGTCGCACTGGCGGTTACGATAAACGCCACTAAAAGTAGATTTACGACCGCCACTATAGA

GCTTCTCTGTACTAGTGTCGCACAGCTAAAACTACATGATGATTGATGTTTTTAAGTGCA

GTTGTGCAAACAAGCCGATTAGTCCTTTCATATATATCTAAGCATTTGACTTTCTGTTAT

CTCTGTCATTAGATGGAGCACGAAACTGATTCTGAAAAAAAAACAGTCAGGTTATCCGGT

TATATATCCCCTCTGAAGAAAAGCTCGTCGTTGATCTTCAGAATTGCTGACCTTGAGCGT

CCATGTGACCATCTATATCCATCTTCGAACGGAGATACATCCGCGCGCGCGGTTCAATGC

ACTCGGTCCACTGCTAGACTGCCTGATGCCTGATCATATAGCAAGCTGCTTGCGGAGCCG

TGCACGCGGCCGCCGTTTTGCAGAATTTGCAGATTTGCATTGCAGGTGTAGCGTACGGCC

GCACCCCACGGCCCACGCGCGACGGACGGGGTCGGGGGCGCAGTACGCACATCTGTCCGC

CGTGTACGTACTCGCGGACACGCGAGTGTACTACCACTGACCACAGTAGTTTCCCTCCGT

CGCGTCGTACTGCACGACGCCCCGCGCTATATATGTAGGCAGCCGCAGAACATGCCCCGT

CCCCGATCGACGCGCACAACACAGCGCCCACACACACACAGACAGTCTTACGCGACGACA

CACACGCAAGGCGGACGGAC

COPT3

ATCAAGCACAAGATCAAAAACAATTTGCACTGATGGTTAAATTAAGAAAAACTGAAAGCC

ATCTAAACACGTATCTCTAAGACACCATATTATTTACAGTCACCAAGTATTGGAATAAAG

GTGAGAGAAGTATTTTCATGCAAAGGTGTCAAACATGATGCATCAATCAGAATGCATGCT

CGTCCTATTCGTCCTCACAAGTTTTGCCAACATAATGTGGCAATAGCGTTCTATATCGGT

GGCATACTTATGACTCTCTCGATATTTTGCTAGTCGTCAGCTACACATACGTTTTCGAGG

TTAGTGTACCTGCAGAAAATTCCATCACAGCCCATTTTTCCCATGATGCAATTCAAACAT

GACAGTTTATCACAGTTTATACTCCATATATTTCTATATGGAGGCTAGCTCTTCATTAAG

CGTCGAGCCACGCATAGGAGCCGCTGCCACACTATAATCATAGGGCAGCCCATTATGGTA

ACTCACTTTTTTACCTGAGCGTAGGTGCGTCGTTGCGAGTACATTACACTTCTCAGTATT

CCCATGTATGTATACCCATACACATCCATGGGGTACTGAATCCCAAAAGTAATTACTCCA

CATCGCAACATTCATATATACATATGTGTAACATGTATATAATCAAGCGCCACAATTAAG

CACTTCCCTAGACCAACGTTTGTTCGGTCATGCTCTCACATCTCACGTTACCTGAGTGTC

GATCCATTCAATACTGAATGGCTTCAAATACAACATTACACATGTATGCAATATCCCCAG

GTTGTGGGTTAGTGTTTTAATCTAAGCCACCTGATAGTCCTTAATTTAGGGCTTAACAAA

GGATGTCGCTGGCATTGTAGTTTTCAAATCTGTAGTTTTTCAAAACCAAACAATGTGTTT

AGTTGAAAATAGGTTTTCCAAAACCAAAACTTTGTTTTGAAAATACATATGTGATCGTAT

TATACTTAATCCTGCTCCGATACCAGCTGTGGCAGCCAAGTTATTGGGCCCACATGCACC

TGCCCTTGTCTCAAAGACCTCATACGGCTACGCATGTGCACTAGATAACTTAACAGGATT

CGTCCGAGTGTTCCAAGGACCCCGGATAAACCAGTTACAACCAGGATCGCAAGATTAAGT

AAACACAAATCACACACCAACATTTTGCAGTGGAATTTCTTTATTACAAAAAGTTACAAG

TTACATTAAGATTACATTATACAAGATCGGAGTGATTATAAAATAAATTCAAAGTTTTTC

ACTTTGAATGATGTATAATTTTTATAAGTTTAAAATACATGCTAGCTTAAGTGACCATCC

TCAAATAGAAGTATAGAAGAGCTACTTATACCTATAAGAAGGTCGTGCCCATCGGCGCTT

AGCACCATCCACAGCAGCACAAATCTTATCTAGAATGGATTGATTGTTCAAACAAAACAC

CCTATAAATTCCAACAGCGTGAAAAGATTTTTTTATAGTATGATGCCCAATTAAGAATTT

AAATTCGGTTGGACTGATTATAACGAACAGTTTACAGCTCTGTTTGACGTAGACCTCCTT

CCCCGACATGCCCGCCACCTTATAGCGTGGATCTTTATTGGCAGATGGCGACCAAAACAA

CCCAAATAATTTAGCAGAAAAGACAAGTGCAATGAACCCTTCATTTACAACAACATAGTG

GTGCTCCGGAATAATTGTCTCTGTATTCGTCTCCGTTTGCTTACCTTAAACACACCGCCA

CCTAGGCCCCAAACAAGTTCATCTCTAAAGCACACGCGACTAAACCAAGAACCTGCTTAC

CTGTTCCTCGTTCTTGTCGCTATCCATTTCTTTCAATTCCACAGAGAACATATACCGCTT

TCCACAACTCGCGGCAACCTCCTCCTCCTTCTCTCTTCCTATTTACTAGCTCATGTGGTG

AACTAGCAAGTTGCAATCACTCTACCACAGCGACCCTTGTCACCAATTAAGAAAGTACTA

GCGGTCCAGAGACAGACGCG
