# Supplementary material for: Identification and functional characterization of the ZmCOPT copper transporter family in maize
Source: PLoS One. 2018 Jul 23;13(7):e0199081. doi: 10.1371/journal.pone.0199081 (PMC6056030; doi:10.1371/journal.pone.0199081)
Supplement: S1 Table — (DOCX) [file pone.0199081.s003.docx]

| Name | Gene ID |
| --- | --- |
| OsCOPT1 | ADP37431.1 |
| OsCOPT2 | XP_015647285.1 |
| OsCOPT3 | XP_015630711.1 |
| OsCOPT4 | BAH92657.1 |
| OsCOPT5 | XP_015638992.1 |
| OsCOPT6 | XP_015649606.1 |
| OsCOPT7 | XP_015651379.1 |
| AtCOPT1 | NP_200711.1 |
| AtCOPT2 | NP_190274.1 |
| AtCOPT3 | NP_200712.1 |
| AtCOPT4 | NP_850289.1 |
| AtCOPT5 | NP_197565.1 |
| AtCOPT6 | NP_850091.1 |
| hCtr1 | NP_001850.1 |
| hCtr2 | NP_001851.1 |
| ScCtr1 | NP_015449.1 |
| ScCtr2 | NP_012045.3 |
| ScCtr3 | NP_013515.3 |
| SpCtr4 | NP_587968.1 |
| SpCtr5 | NP_594269.1 |
| SpCtr6 | NP_595861.1 |
| ZmCOPT1 | XP_020396846.1 |
| ZmCOPT2 | NP_001152680.2 |
| ZmCOPT3 | XP_008679629.1 |
