# Supplementary material for: Identification and functional characterization of the ZmCOPT copper transporter family in maize
Source: PLoS One. 2018 Jul 23;13(7):e0199081. doi: 10.1371/journal.pone.0199081 (PMC6056030; doi:10.1371/journal.pone.0199081)
Supplement: S2 Table — (DOCX) [file pone.0199081.s004.docx]

| *ZmCOPT1* | | *ZmCOPT2* | | *ZmCOPT3* | |
| --- | --- | --- | --- | --- | --- |
| cis-elements | function | cis-elements | function | cis-elements | function |
| AAGAA-motif | GAAAGAA | A-box | cis-acting regulatory element | ABRE | cis-acting element involved in the abscisic acid responsiveness |
| AE-box | part of a module for light response | ABRE | cis-acting element involved in the abscisic acid responsiveness | ARE | cis-acting regulatory element essential for the anaerobic induction |
| ARE | cis-acting regulatory element essential for the anaerobic induction | ARE | cis-acting regulatory element essential for the anaerobic induction | ATCT-motif | part of a conserved DNA module involved in light responsiveness |
| ATCT-motif | part of a conserved DNA module involved in light responsiveness | AT-rich element | binding site of AT-rich DNA binding protein (ATBP-1) | Box I | light responsive element |
| ATGCAAAT motif | cis-acting regulatory element associated to the TGAGTCA motif | ATGCAAAT motif | cis-acting regulatory element associated to the TGAGTCA motif | CAAT-box | common cis-acting element in promoter and enhancer regions |
| Box 4 | part of a conserved DNA module involved in light responsiveness | Box 4 | part of a conserved DNA module involved in light responsiveness | CATT-motif | part of a light responsive element |
| Box I | light responsive element | Box I | light responsive element | CG-motif | part of a light responsive element |
| CAAT-box | common cis-acting element in promoter and enhancer regions | Box III | protein binding site | CGTCA-motif | cis-acting regulatory element involved in the MeJA-responsiveness |
| CAT-box | cis-acting regulatory element related to meristem expression | Box-W1 | fungal elicitor responsive element | CTAG-motif |  |
| CATT-motif | part of a light responsive element | CAAT-box | common cis-acting element in promoter and enhancer regions | G-box | cis-acting regulatory element involved in light responsiveness |
| E2Fb |  | CAT-box | cis-acting regulatory element related to meristem expression | GAG-motif | part of a light responsive element |
| G-box | cis-acting regulatory element involved in light responsiveness | CCGTCC-box | cis-acting regulatory element related to meristem specific activation | GT1-motif | light responsive element |
| GA-motif | part of a light responsive element | CGTCA-motif | cis-acting regulatory element involved in the MeJA-responsiveness | HSE | cis-acting element involved in heat stress responsiveness |
| GATA-motif | part of a light responsive element | CTAG-motif |  | LAMP-element | part of a light responsive element |
| I-box | part of a light responsive element | G-box | cis-acting regulatory element involved in light responsiveness | MBS | MYB binding site involved in drought-inducibility |
| MBS | MYB binding site involved in drought-inducibility | GAG-motif | part of a light responsive element | MBSII | MYB binding site involved in flavonoid biosynthetic genes regulation |
| P-box | gibberellin-responsive element | GARE-motif | gibberellin-responsive element | MNF1 | light responsive element |
| Skn-1_motif | cis-acting regulatory element required for endosperm expression | LTR | cis-acting element involved in low-temperature responsiveness | Skn-1_motif | cis-acting regulatory element required for endosperm expression |
| TATA-box | core promoter element around -30 of transcription start | MBS | MYB binding site involved in drought-inducibility | TATA-box | core promoter element around -30 of transcription start |
| TATCCAT/C-motif |  | MBSII | MYB binding site involved in flavonoid biosynthetic genes regulation | TC-rich repeats | cis-acting element involved in defense and stress responsiveness |
| TC-rich repeats | cis-acting element involved in defense and stress responsiveness | MRE | MYB binding site involved in light responsiveness | TGA-element | auxin-responsive element |
| TCA-element | cis-acting element involved in salicylic acid responsiveness | Skn-1_motif | cis-acting regulatory element required for endosperm expression | TGACG-motif | cis-acting regulatory element involved in the MeJA-responsiveness |
| TCT-motif | part of a light responsive element | TATA-box | core promoter element around -30 of transcription start | chs-CMA1a | part of a light responsive element |
| ACE | cis-acting element involved in light responsiveness | TC-rich repeats | cis-acting element involved in defense and stress responsiveness | AAGAA-motif |  |
| EIRE | elicitor-responsive element | TCA-element | cis-acting element involved in salicylic acid responsiveness | GARE-motif | gibberellin-responsive element |
| MNF1 | light responsive element | TGA-element | auxin-responsive element | GATA-motif | part of a light responsive element |
| Sp1 | light responsive element | TGACG-motif | cis-acting regulatory element involved in the MeJA-responsiveness | I-box | part of a light responsive element |
| TCCACCT-motif |  | W box |  | TATCCAT/C-motif |  |
| TGA-element | auxin-responsive element | chs-CMA1a | part of a light responsive element | TCA-element | cis-acting element involved in salicylic acid responsiveness |
|  |  | Pc-CMA2c | part of a light responsive element | TGACG-motif | cis-acting regulatory element involved in the MeJA-responsiveness |
|  |  |  |  | circadian | cis-acting regulatory element involved in circadian control |
