# Supplementary material for: Identification and functional characterization of the ZmCOPT copper transporter family in maize
Source: PLoS One. 2018 Jul 23;13(7):e0199081. doi: 10.1371/journal.pone.0199081 (PMC6056030; doi:10.1371/journal.pone.0199081)
Supplement: S3 Table — (DOCX) [file pone.0199081.s005.docx]

| ZmCOPT1-F | CTCGGCTACCTGCTCAT |
| --- | --- |
| ZmCOPT1-R | GAACAAGGAAGGCAAACC |
| ZmCOPT2-F | ACGGCAAGGATGAGGTC |
| ZmCOPT2-R | CCAAACAGCAACACGGA |
| ZmCOPT3-F | GTAACGGTGATGTCCCTT |
| ZmCOPT3-R | TTCCTCCGCAAATGACAG |
