# Supplementary material for: Identification and functional characterization of the ZmCOPT copper transporter family in maize
Source: PLoS One. 2018 Jul 23;13(7):e0199081. doi: 10.1371/journal.pone.0199081 (PMC6056030; doi:10.1371/journal.pone.0199081)
Supplement: S4 Table — (DOCX) [file pone.0199081.s006.docx]

| ZmCOPT1-F | CGGAATTCAGTCCCAACCGTTCCCT |
| --- | --- |
| ZmCOPT1-R | CGGGATCCAATCCGGCGACCCCATA |
| ZmCOPT2-F | CGGGATCCCAAGGCGGACGGACATG |
| ZmCOPT2-R | CGAGCTCGACGAGCCTAACAGCACACG |
| ZmCOPT3-F | CGGAATTCACGCGATGGCGACGATG |
| ZmCOPT3-R | ACGAGCTCTCGAGAAGAGACTCAAGGCT |
