# Supplementary figures and images for: Identification and functional characterization of the ZmCOPT copper transporter family in maize
Source: PLoS One. 2018 Jul 23;13(7):e0199081. doi: 10.1371/journal.pone.0199081 (PMC6056030; doi:10.1371/journal.pone.0199081)

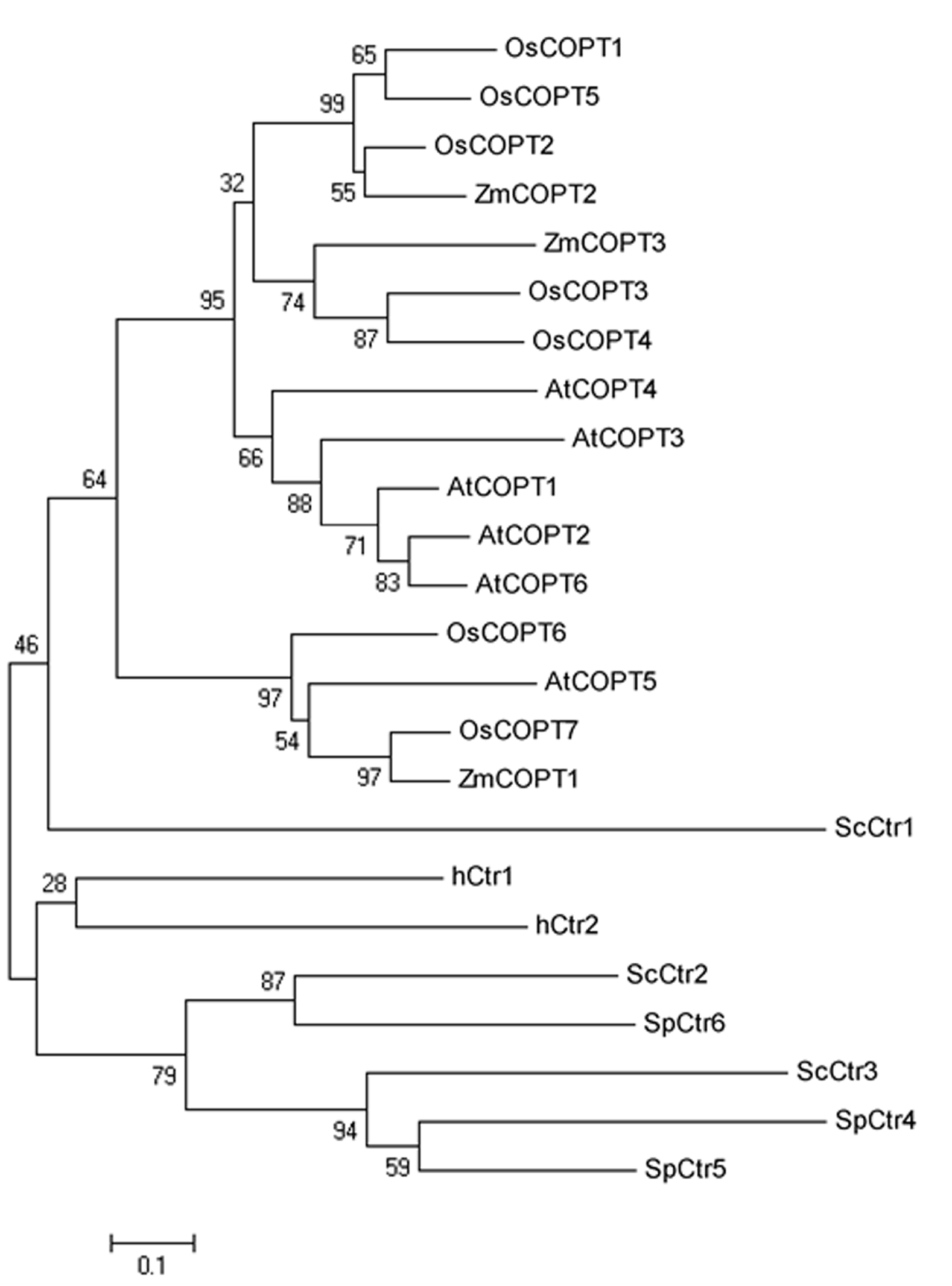

Supplement: S2 Fig — (TIF) [file pone.0199081.s009.tif]

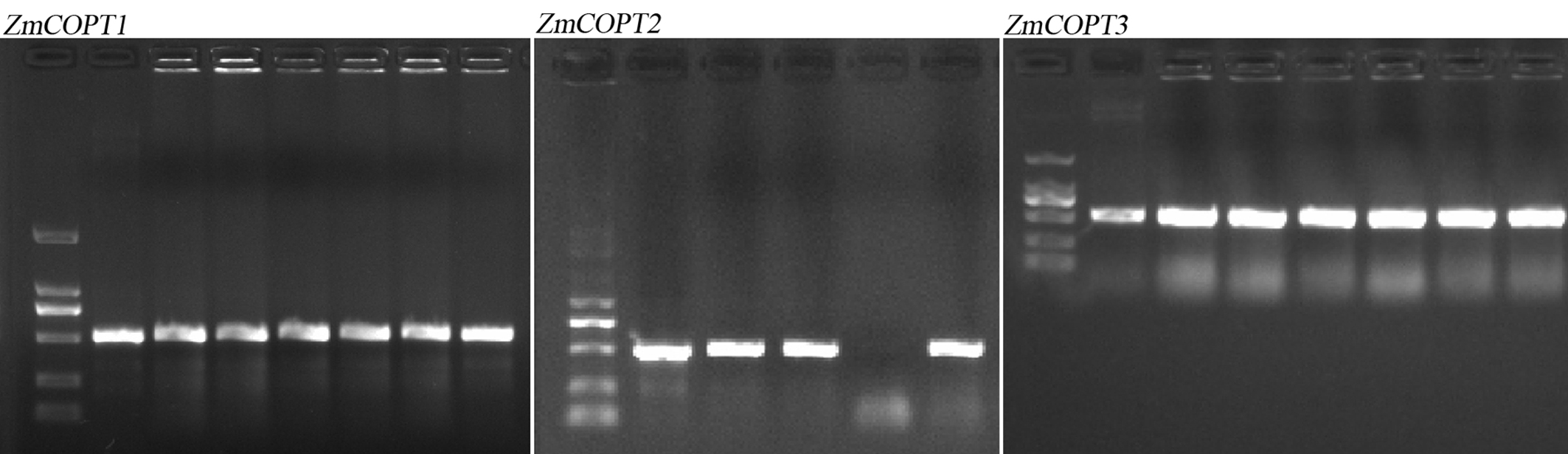

Supplement: S4 Fig — (JPG) [file pone.0199081.s011.jpg]
